# Driving macro-scale transformations in three-dimensional-printed biopolymers through controlled induction of molecular anisotropy at the nanoscale

**DOI:** 10.1098/rsfs.2023.0077

**Published:** 2024-06-07

**Authors:** Laia Mogas-Soldevila, Jorge Duro-Royo, Daniel Lizardo, George G. Hollyer, Charles M. Settens, Jordan M. Cox, Johannes T. B. Overvelde, Elaine DiMasi, Katia Bertoldi, James C. Weaver, Neri Oxman

**Affiliations:** ^1^ DumoLab Research, University of Pennsylvania, Philadelphia, PA 19104, USA; ^2^ Mediated Matter Group, Massachusetts Institute of Technology, Cambridge, MA 02142, USA; ^3^ Department of Materials Science and Engineering, University of Pennsylvania, Philadelphia, PA 19104, USA; ^4^ MIT.nano, Massachusetts Institute of Technology, Cambridge, MA 02139, USA; ^5^ Soft Robotic Matter, AMOLF Institute, Amsterdam 1098, The Netherlands; ^6^ Lawrence Berkeley National Laboratory, Berkeley, CA 94720, USA; ^7^ School of Engineering and Applied Sciences, Harvard University, Cambridge, MA 02138, USA; ^8^ Wyss Institute for Biologically Inspired Engineering, Harvard University, Boston, MA 02215, USA; ^9^ Oxman, New York, NY 10019, USA

**Keywords:** molecular anisotropy, self-folding, biological materials, additive manufacturing, multiscale design, large-scale structures

## Abstract

Motivated by the need to harness the properties of renewable and biodegradable polymers for the design and manufacturing of multi-scale structures with complex geometries, we have employed our additive manufacturing platform that leverages molecular self-assembly for the production of metre-scale structures characterized by complex geometries and heterogeneous material composition. As a precursor material, we used chitosan, a chemically modified form of chitin, an abundant and sustainable structural polysaccharide. We demonstrate the ability to control concentration-dependent crystallization as well as the induction of the preferred orientation of the polymer chains through the combination of extrusion-based robotic fabrication and directional toolpathing. Anisotropy is demonstrated and assessed through high-resolution micro-X-ray diffraction in conjunction with finite element simulations. Using this approach, we can leverage controlled and user-defined small-scale propagation of residual stresses to induce large-scale folding of the resulting structures.

## 1. Introduction

There is a growing interest within both design and engineering communities to develop large-scale self-organizing materials and structures displaying programmable folding, curling or shaping phenomena [1–3]. Motivated by the opportunities and potential impact associated with this challenge, we harness a design and digital fabrication platform that attempts to link architectural-scale three-dimensional folding with the induction of anisotropy at the molecular scale. Using this approach, we observe a direct relationship between the preferred orientation of polymer chains and their programmed extrusion in metre-scale 2.5-dimensional printed geometries. These discoveries help to lay the groundwork for the development of new approaches to the control hierarchical assembly of complex structures, with the potential to exhibit versatile mechanical properties and environmental adaptability that match those often found in biologically grown structures [2,4,5].

### 1.1. Polymer orientation

The ability to precisely tune both organization and composition of polymeric materials can provide for a wide range of desirable properties and behaviours at both product and architectural scales. For example, it has been well documented in the synthetic polymer manufacturing community that chain alignment and crystallinity can be leveraged for the tunability of mechanical properties or the induction of self-folding phenomena [6–11]. Similarly, the introduction of crystalline order within naturally derived biopolymers poses a great opportunity to enhance material performance for the design and digital fabrication of new classes of composite structures [12]. The main challenge associated with the induction of organizational order across spatial and temporal scales; however, is the relative lack of robust techniques for arranging biopolymer molecular building blocks into hierarchical structures, given a desired performance. To achieve these goals, researchers frequently employ both physically and chemically guided techniques that harness the self-assembling properties of biopolymers into ordered structures over dimensions spanning from the nanoscale to the microscale [12,13]. Using these approaches, researchers have been successful in ordering the internal structures of several biopolymers, including collagen gels, collagen–alginate blends and regenerated silk fibroin [14–16]. Methods to achieve these results have included methanol treatment of films, direct or anisotropic aqueous straining of gels (i.e. compressing or stretching) [17,18] and crystalline mesophases [19], as well as the application of uniaxial extension [15] and external electric fields over biopolymer blends [20,21], or electrospinning of nanofibres [22].

### 1.2. Self-folding in natural systems

Form modulation often occurs in biological systems in response to environmental forcing [1,23], through the cooperative integration of shape, structure and material—as well as through biologically hardcoded processes operating *simultaneously* within organically grown tissues [4,23,24]. Bending, folding and other such self-shaping forms in these cases often occur owing to the presence of residual internal stresses rather than through the application of externally imposed mechanical stresses [2]. These pre-programmed biological strategies for achieving shape *tunability* include (i) the controlled orientation of material reinforcement, (ii) the asymmetric distribution of material reinforcement, and (iii) the breakdown and reorganization of material reinforcement [1]. Because the first two strategies include environmentally responsive materials that frequently do not require the input of cellular energy (as described below), they provide fertile ground for research on programmable matter and active technical composites [25].

Inspired by this potential, there is an active search in the design and engineering sciences to create materials and structures with self-arranging capabilities in response to environmental stimuli, leveraging strategies observed in biological structures across scales [9,26–32]. For example, at the *nanoscale,* DNA self-assembles into helices [2] using chiral twisting, while linear polypeptide chains fold to form three-dimensional proteins with different structures, properties and functions [30,31]. At the *micro-scale,* helical self-shaping occurs in climbing plant tendrils and orchid tree seed pods using passive means in search for minimum energy configurations driven by material reinforcement within inner fibre architectures [1,33]. Water also plays an important role in plant self-shaping [34,35], with folding by shrinking and swelling of plant tissue in leaves, stems and roots driven by a close interplay between internal water content and cell turgor pressure [35]. The regulation and coupling of these processes, in turn drives the deformation of tissue at different levels of hierarchy using both microscale geometrical constraints and nanoscale polymer chemistry [25]. Without the use of metabolic energy, pre-programmed hygro-morphs in the dying tissue of pine cones mechanically open owing to local changes in water availability [1,24,36]. At the *mesoscale,* nemertean and turbellarian worms achieve passive shape change using fibrous lattice rearrangements in their epidermal membranes [37], and in direct response to chemical stimuli, echinoderms such as sea cucumbers are able to modify the interactions between their dermal collagen fibres to change their overall stiffness on timescale of a few seconds [1]. At the *macro-scale,* trees are able to tune their cellulose microfibril angle based on environmental cues such as gravity, wind or damage, deriving heterogeneous internal architectures that regulate bending and overcome load stresses in branches [1].

### 1.3. Self-folding in synthetic systems

Inspired by biological examples such as those described above, research on analogous synthetic systems that can morph or self-fold in a controllable manner points towards a wide range of applications across scales—from microscale biomedical devices, to macroscale aircraft components [38]. Current material and fabrication techniques for induced folding can be classified according to their relative scale and application domains. At the *nanoscale*, DNA origami workflows have been developed for the precise and tailored folding of single DNA strands into complex three-dimensional geometries [39], echoing traditional origami techniques that enable the creation of complex shapes from a series of simple paper folds [40]. On the *micro-scale*, precise microengineering of smart materials via the self-folding of thin polymeric films into three-dimensional geometries is enabled by using specific stimuli such as light, heat, pH, electric fields or chemical gradients [41–43]. As reviewed in [38], bio-inspired shape transformation in soft materials by modulating internal stresses includes programmed folding of bilayers, the evolution of three-dimensional configurations from non-equilibrium states, differential cross-linking, halftone lithography, small-scale modulation of stresses, iono-printing via electrochemical electrodes, electronically programmable three-dimensional hydrogels and inorganic nanomaterial composites to induce shaping [38]. On the *mesoscale*, the production of functional robots via the induced folding of two-dimensional plans into three-dimensional shapes using joule heating actuation or through the design of complex pop-up-book-like mechanisms has been demonstrated [44].

Laser-engraved origami with actuated hinges or active spring elements have also been used for the production of mesoscale self-assemblies [23]. In addition, ‘four-dimensional’ printing technologies have been developed that incorporate time-dependent shape-changing geometries and direct multi-material printing or printing and casting for the production of active composites that can react to environmental stimuli [9,10,33,45]. To achieve these effects, four-dimensional printing frequently uses composite inks that inform elastic and swelling anisotropies; as well as dynamic materials such as thermo-responsive shape memory fibres or hydrophilic hinges within flexible matrixes [9,30,33,45]. Balsa-wood-inspired three-dimensional printing of lightweight cellular composites, for example, is enabled by the high aspect ratio fibre reinforcement within the three-dimensional printing inks. Here, orientation of mesoscale fibrils takes place at the printing nozzle under shear and extensional flow fields [46]. On the *macro-scale*, based on the moisture-sensitive characteristics of wood, weather-responsive self-folding of macro-scale architectural components and assemblies can be designed from simple wood elements [47] or by three-dimensional-printing custom wood grain structures from single wood fibres or multi-material combinations [48]. Such hygroscopic actuation methods harness natural swelling and shrinking present in the cellular structure of wood, which provides a dimensional shape change of up to 10% perpendicular to the grain [49]. Recently, large-scale warping and morphing techniques have been used to control the surface of aircraft wings and adaptively change their chamber geometry during flight through the incorporation of actuated elastomeric materials [50]. These constant adjustment methods allow for increased flight efficiency at multiple altitudes, reduction of structural weight and improved fuel economy, as well as decreased environmental and noise impacts [50,51].

Despite these advances, additional research is required to better understand shape change and self-folding driven by natural microstructural features, as man-made solutions often remain less effective than their biological counterparts. For example, in bio-inspired self-shaping materials triggered by hydration, there are still many unknowns and possibilities regarding the directed optimization of energy efficiency, morphological reversibility and shaping accuracy [1], as well as the discovery of simple techniques for complex shape control. Towards this goal, the present study describes a digital design and fabrication methodology and a predictive material simulation model, using water-based biological materials and inducing two-dimensional-to-three-dimensional non-reversible anisotropic self-folding within single-material manufactured objects across multiple length scales. Our manufacturing system is able to operate at the nanoscale (via molecular chain alignment and the induction of crystallinity), micro-scale (via directional extrusion design), mesoscale (via global stress line toolpaths) and macro-scale (via architectural design), demonstrating high degrees of opportunity to tune and control both material properties and global geometric shape.

## 2. Material and methods

### 2.1. Overview of water-based digital fabrication platform

**Figure 1.**
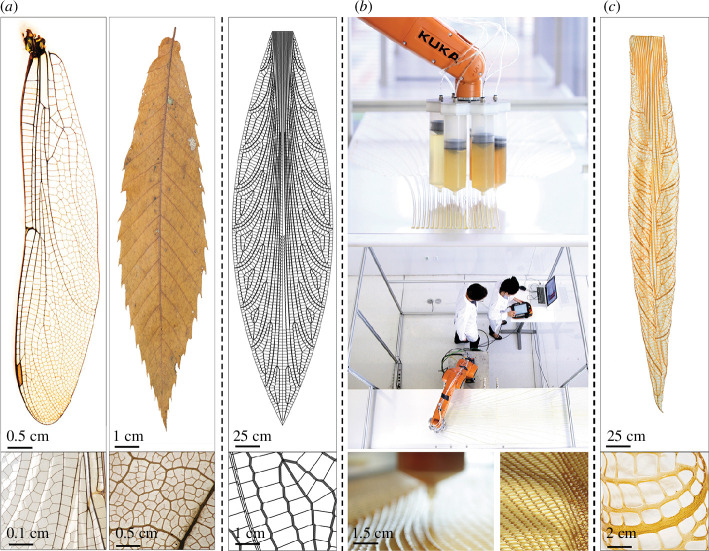
Overview of the WDFP and specific examples of manufacturable geometries. (*a*) INSPIRATION: biomimetic case study digital design process for 2.5-dimensional toolpath generation based on dragonfly wing and leaf venation patterns of a 3-cm-long dragonfly wing specimen and a 10 cm-long dried leaf specimen. (*a*, right) VIRTUAL: 3 m-long digitally generated toolpath with hierarchical geometric patterning. (*b*) PHYSICAL: Fabrication platform features: robotic arm positioning system with a 1 m reach and 10 kg payload and multi-nozzle pressure deposition system carrying diverse chitosan hydrogel concentration ranging from 2% to 12% w/v in 4% acetic acid. (*c*) RESULTS: 3 m-long self-folded column cured at ambient conditions and a magnified view of structural folding along toolpaths induced through the fabrication process.

The water-based digital fabrication platform (WDFP) (figure 1) is a three-component additive manufacturing extrusion method that operates at ambient conditions and consists of (i) a computational model, (ii) an enabling technology, and (iii) a design-to-production workflow adaptable to a wide range of water-based low-viscosity materials for the production of large-scale 2.5-dimensional multi-material constructs that are cured at room temperature via evaporation [52,53]. Specifically, every 2.5-dimensional-printed layer in the WDFP can be independently defined in terms of: differentially distributed polymer concentration in aqueous solvents or in water suspensions (as demonstrated in [54,55]); variable motion speed of nozzle positioning; and tailored flow rates to desired extrusion cross-section thickness along paths [52]. In the context of the present study, the term ‘2.5-dimensional’ is used to describe geometries that encompass relatively simple vertical translations of an initially planar (two-dimensional) geometry.

### 2.2. Fabrication software

Object designs and toolpaths for the WDFP were created using the CAD modelling environment Rhino3D (Rhinoceros, Robert McNeel and Associates, USA) and its parametric programming plug-in, Grasshopper. Its geometric kernel library aids in the production of C-sharp code to transmit fabrication XML instructions to an interface written in C++ using Qt, an open-source programming platform (Qt Project, Norway). It processes input and output data from the design environment, to and from mechanical parts. Transmission to the robotic arm positioning system was performed via an Ethernet UDP socket, and via a serial USB signal to the deposition system. The pneumatic tool firmware was developed in C code using the Eclipse IDE environment (The Eclipse Foundation, Canada) and the cross-platform open-source Arduino library (Arduino Software, Italy) [52,56,57].

### 2.3. Fabrication hardware

A pneumatic hardware assembly provided positive and negative pressure controlled serially through a micro-board and a relay shield (Arduino Mega 2560). Directional three-way two-position solenoid valves controlled and distributed pressure to material barrels and pressure was tuned with an electronic pressure regulator, converting a 4–20 mA signal. The signal to the regulator was computed through a low-power high-accuracy digital-to-analogue converter board with non-volatile memory. For motion means, a six-axis robotic arm (Kuka KR AGILUS) was used to describe defined toolpaths and to precisely position a customized assembly of pressurized material barrels in three dimensions.

As previously described in [53,58], a customized pneumatic deposition system was attached to the end-effector of an existing Kuka KR AGILUS robotic arm positioning platform. At the end-effector, materials were contained within six 300 ml plastic dispensing syringe barrels equipped with plungers. Extrusion nozzle diameter ranges from 1 to 7 mm and was custom fabricated from HDPE plastic. Pneumatic circuitry for flow valves, gauges and tubing was dimensioned for a range of 10–120 psi. Positive pressure was obtained through a portable air compressor with 1.5 hp, 120 V, 60 Hz 4.2 cfm at 90 psi and 5.4 cfm at 40 psi. Negative pressure was obtained with a rotary vacuum pump at 1725 rpm, 110 volts and 60 Hz.

### 2.4. Materials

Eighty-five per cent deacetylated high molecular weight chitosan and 90% glacial acetic acid were purchased from Sigma Aldrich. Chitosan blends at different concentrations (2–12% w/v) were dissolved in 4% w/v acetic acid aqueous solution. These different formulations displayed viscosities ranging from 500 to 50 000 cPs and exhibited visco-elastic and visco-plastic behaviour when contained within airtight barrels. These different blends underwent extrusion and curing at room temperature [52]. Meta-data on materials were stored in the platform’s database to inform calculations within the computational model’s parameters for the WDFP’s deposition and positioning. Their testing, characterization and processing are explained in detail in previous publications and a patent [53,58,59].

### 2.5. Sample printing and characterization

**Figure 2.**
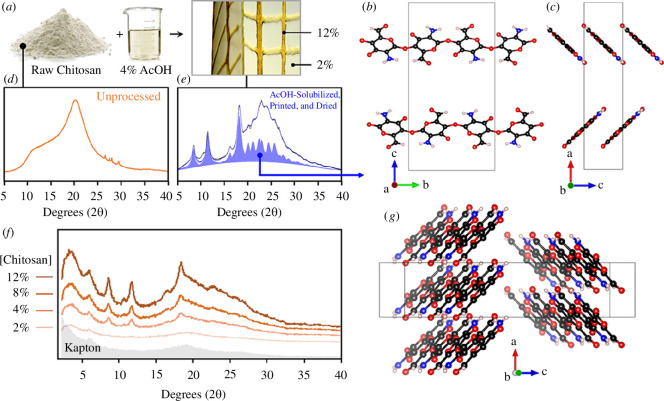
Material formulation and molecular-scale crystallographic characterization (*a*) Pictorial representation of our material preparation strategy: through the solubilization of different concentrations of raw chitosan powder in acetic acid, we can create a wide range of material viscosities that can be extruded for the production of complex 2.5-dimensional architectures (cf. figure 1). (*d*) X-ray diffraction pattern (XRD) (Rigaku Miniflex) of raw unprocessed chitosan. (*e*) XRD pattern (Rigaku Miniflex) of chitosan after solubilization in AcOH, printing and drying. The solid blue pattern represents the Pawley fit of an α-chitosan structure, which was subsequently used to generate the proposed crystal structure of α-chitosan shown in (*b–g*). (*f*) Powder XRD (Argonne APS 11BM) analysis of dried acetic acid-solubilized chitosan films (of equal mass), demonstrating a clear correlation between polymer concentration (2–12%) and the extent of crystallinity. The background diffraction pattern obtained from the Kapton capillary sample holder is highlighted in grey.

**Figure 3.**
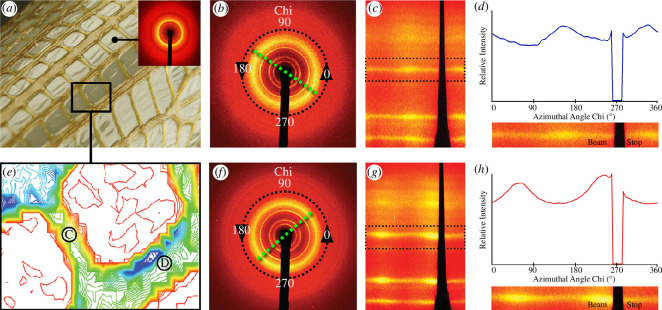
Directional anisotropy of 2.5-dimensional-printed chitosan-based constructs. (*a*) Photograph of a 2.5-dimensional-printed chitosan ribbed structure with an inset diffraction pattern corresponding to the cast 2% chitosan film. (*e*) X-ray transmission intensity plot used for identification of the regions of interest at the synchrotron beamline (from red to green to blue, the colour scale corresponds to relative sample thickness). Left rib C: 12% w/v chitosan in 4% w/v acetic acid, 2 mm inner diameter nozzle and two layers in height and one in width; Right rib D: 12% w/v chitosan in 4% w/v acetic acid, 2 mm inner diameter nozzle and three layers in height and six in width (parallel extrusion). (*b*,*d*) XRD patterns acquired from ribs C and D in panel (*e*). Images are scaled yellow at the most intense reflections. Green dashed lines indicate the azimuthal direction of maximum diffraction intensity. For both ribs, this line is parallel to the rib direction. (*c,g*) Diffraction patterns of (*b,f*) shown with azimuthal chi variable ‘unrolled’ along the horizontal axis. (*d*,*h*) Intensities (*b,f*) integrated within a–q interval around the intense reflection and plotted versus chi. The data reveal that the preferred orientation of the chitosan crystallites follows the direction of each rib.

**Figure 4.**
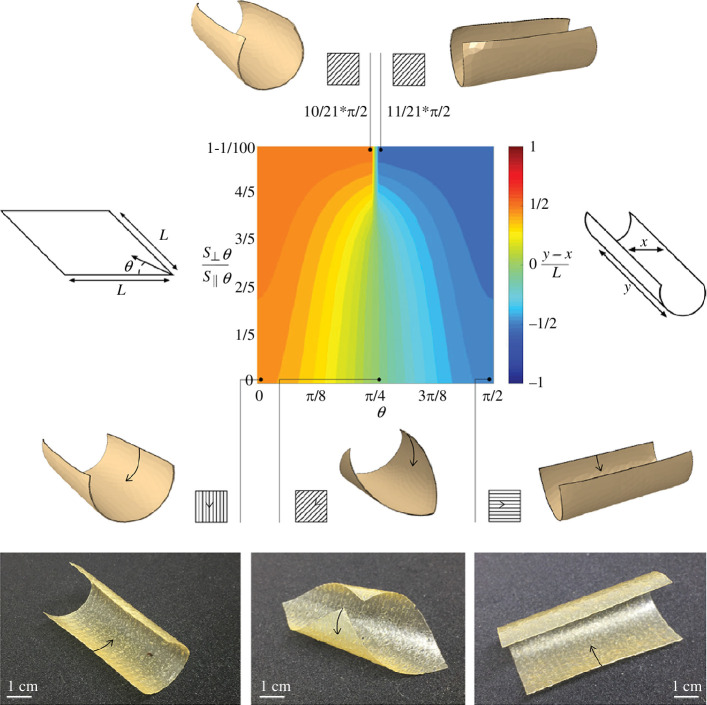
Numerical simulations and experiments of fabrication-induced folding as a function of structural anisotropy. *Top*: contour plot showing the dependency of the folding behaviour on the direction of anisotropy θ, and the size of anisotropy introduced by varying the swelling ratio parallel and orthogonal to θ, $\left(\frac{S⊥\theta }{S||\theta }\right)$ . The folding direction is characterized by the absolute change in distance between the outer points lying on the short axis of the square. *Bottom*: experimental verification of the numerical model, showing a relatively high level of anisotropy (i.e. $\frac{y-x}{L}$ ≥ $\frac{\surd 2}{L2}$ ), as evidenced by the direct link between print direction and folding direction (noted with arrows on two- and three-dimensional schematics and on photographs of shaped constructs).

To identify the factors driving the often non-intuitive folding geometries observed in figure 1, we designed a series of simplified experiments to investigate the impacts of the material extrusion process on the introduction of molecular anisotropy. For these studies, all analysed samples were printed using the extrusion hardware and software described above. The samples analysed in figure 2 were cast as small rectangular swatches using 2, 4, 8 and 12% w/v chitosan in 4% w/v acetic acid, respectively. The sample in figure 3 was fabricated by printing high-concentration chitosan (12% w/v chitosan in 4% w/v acetic acid) parallel to the rib direction (dark brown) and by casting low concentration (2% w/v chitosan in 4% w/v acetic acid) chitosan in the skin pockets (light brown). Square samples shown in figure 4 had tool-paths parallel and diagonal to the side, respectively. All constructs were fabricated and handled at ambient conditions, and left to dry overnight under a large array of speed-controlled fans [52] to permit uniform solvent evaporation. The sample was then mechanically removed from the substrate using metal or plastic spatulas, and allowed to freely self-fold. Since the samples were not coated with water-proofing agents, the monitoring of ambient humidity was critical to ensure that the samples remained partially flexible (and crack-free) at the time of removal.

Powder X-ray diffraction (XRD) analyses of dried acetic acid-solubilized chitosan samples at different concentrations (figure 2) were performed on cast thin films and raw un-solubilized material powder. The resulting material samples were ground under liquid nitrogen, and equal masses of each of the different powdered samples were either analysed with a Gen 6 Rigaku Miniflex at a wavelength of 1.5406 Å, step size of 0.02° and scan rate of 2.3° min^−1^, or placed into kapton capillaries and analysed at the 11BM beamline at the Argonne Advanced Photon Source (with an electron energy of 7 GeV with a critical photon energy of 19.5 keV). The samples were scanned from 0.5 to 50° 2θ and were calibrated using a mixture of *ca* 70 wt% Si (SRM 640d) and *ca* 30 wt% Al_2_O_3_ (SRM 676). Corrections were applied for detector sensitivity, 2θ offset, small differences in wavelength between detectors and the source intensity, as noted by the ion chamber before merging the data into a single set of intensities evenly spaced in 2θ. All of the acquired XRD patterns (regardless of the X-ray energies employed) were converted into copper *k*-alpha 2θ values to permit direct comparisons. For the different chitosan concentrations, the relative ratios of crystalline and amorphous phases in the dried cast films were estimated by first subtracting the diffracted contribution of the surrounding kapton capillary. For each of the resulting background-corrected diffraction patterns, a flat baseline was created and the areas under the well-defined Bragg reflections from the crystalline chitosan, and the broad amorphous hump were calculated.

For the printed samples (figure 3), synchrotron X-ray data were collected at Beamline X6B at the National Synchrotron Light Source, Brookhaven National Laboratory, using 19 keV X-rays (λ = 0.65 Å) and a beam spot focused to *ca* 100 μm × 100 μm. Printed chitosan samples were mounted onto the beamline sample holder in transmission geometry. Transmitted X-ray intensity values were recorded using a photodiode detector fixed beyond the sample at the beam stop and normalized by incident intensity measured with an upstream ion chamber to produce the density contours shown in figure 3*e*. Diffraction data were acquired with a Princeton Instruments CCD detector approximately 15 cm beyond the sample. The detector pixel positions were calibrated to momentum transfer Q using a sintered corundum standard, per the JCPDS data card, and the software package Datasqueeze. The diffraction data are shown as raw detector images in figure 3*b,f*; corrected for spatial distortions within the detector optical taper and ‘unrolled’ along the chi axis using MatLab for figure 3*c,g*; and the intensity was integrated around the main diffraction ring in the Q interval 1.15–1.35 Å^−1^ using Datasqueeze for figure 3*d,h*. Crystallite size analysis was performed on the Rigaku Miniflex XRD data, taking care to subtract the instrument contribution of breadth with a NIST LaB6 standard 660b, and estimated using the Williamson–Hall method [60].

### 2.6. Finite-element simulations

Numerical nonlinear implicit folding simulations were performed using the finite element analysis code Abaqus (v. 6.12-1) (figure 4). The square sheets were modelled as a bi-layer with a thickness-to-length ratio of *t*/*L* = 0.005, in which both layers had the same thickness. The layers were meshed using one layer of linear three-dimensional solid wedge elements (C3D6). Each layer was modelled using linear elastic material properties with Young’s modulus E (simulation is independent of absolute value), and a Poisson ratio of ν = 0.3. Shrinkage of the top layer was effectively induced by giving the material orthotropic heat expansion coefficients and reducing the temperature. In the simulations, rigid body translation and rotation were constrained, while gravity was not considered.

## 3. Results

In the present study, we employed our novel additive manufacturing process, the WDFP [52], for the production of large-scale hierarchical structures informed by those found in natural systems. In this approach, biological materials such as chitosan, a chemically modified form of chitin (Nature’s second most abundant biopolymer), are deposited along structural paths that describe major load-bearing stress lines. Deposition can be performed longitudinal, transverse or oblique to geometric paths so that different macro-scale structural motifs can be integrated into the final form. One of the key observations when working with this printing platform is that when fully cured, the resulting constructs (figure 1) frequently exhibit non-intuitive non-reversible two-to-three-dimensional curling behaviours. As such, one of the major goals of the present study was to explore the potential mechanisms responsible for the production of these resulting three-dimensional geometries from simple two-dimensional toolpaths. To address these questions, our approach was multifaceted. First, we investigated the effects of concentration-dependent crystallization (figure 2) as well as induction of the preferred orientation of polymer chains (figure 3) within structural paths at the nanoscale using synchrotron X-ray scattering, followed by the development of a finite element-based model for exploring the effects of this resulting structural anisotropy on the induction of directional folding (figure 4).

### 3.1. Fabrication process

Figure 1 depicts the bio-inspired design, fabrication and resulting cured construct of a 3-m-long chitosan-based additively manufactured and self-folded column inspired by a dragonfly wing and leaf venation patterns (figure 1*a*). The WDFP consists of a highly precise six-axis robotic arm positioner, with a multi-nozzle pressure-based extruder as its end-effector, carrying diverse chitosan hydrogel concentrations ranging from 2% to 12% w/v in 4% aqueous solution of acetic acid (figure 1*b*). The platform synchronizes robotic positioning speeds and nozzle feed pressure to define the geometry of the resulting constructs. These parameters are calibrated for each material to achieve well-defined and continuous extrusion geometries and depend on material viscosity. For example, chitosan gel concentrations of 1–3% w/v in 4% acetic acid aqueous solution have low viscosity (12–800 cP), use low pressures of 5–10 psi to be extruded through the nozzle and do not present shear thinning out of the nozzle. Conversely, chitosan gel concentrations of 4–12% w/v in 4% acetic acid aqueous solution have a high viscosity (1500–8500 cP), require higher pressures of 25–45 psi to be extruded through the nozzle and exhibit shear thinning out of the nozzle. Additional details on these metrics can be found in our previous work focusing on the design of this fabrication platform [52,53,59]. At room temperature, and through water evaporation, the structure is cured, displaying a large-scale hierarchically structured symmetrically folded column (figure 1*c*). It should be noted that since the samples are printed on a rough gripping textured aluminium surface, only the height of the samples change during evaporation, and not along the in-plane dimensions. Upon removing the samples from the surface, they self-fold to their final curved state in a matter of minutes and remain folded in this final three-dimensional geometry.

### 3.2. X-ray diffraction

Powder XRD analysis of raw (un-processed) chitosan powder reveals a largely amorphous signature (figure 2*d*), consistent with data reported in previous studies [61–64]. Upon solubilization in acetic acid, followed by extrusion and drying, the chitosan material adopts a distinctly crystalline compositional signature (figure 2*e*), with an estimated average crystallite size of 10 nm from analysis of the 002, 012 and 101 reflections. Further analysis of this diffraction data was used to construct the proposed chitosan crystal structure shown in figure 2*b–g*. Exhibiting structural features similar to that of α-chitin, chitosan exhibits an ordered fibrillar twofold helix structure with the polymer chains oriented along the *b*-axis of the unit cell [56,65]. The crystal packing is formed from a zig-zag-like organization of anti-parallel chitosan chains (figure 2*b,c,g*); however, unlike α-chitin, chitosan chains can also adopt several distinct conformations, including a variety of helical conformations affected by the level of acetylation [65,66]. Our proposed crystallographic phase identification of the processed chitosan assumes an α-chitin structure, which was de-acetylated and fit using Pawley fit routine to the chitosan crystal model, with a P 21 21 21 (no. 19) space group, and the following unit cell measurements: *a =* 5.005 Å, *b =* 11.141 Å, *c =* 21.650 Å, α *=* 90°, β *=* 90°, γ *=* 90° (figure 2*e*).

Synchrotron powder diffraction measurements performed on printed or cast samples ranging from 2% to 12% chitosan concentration demonstrated a clear link between chitosan concentration and the degree of crystallinity (figure 2*f*). Since equal masses of each of the resulting dried and powdered samples were analysed, the documented increase in crystallinity as a function of printed concentration could not simply be attributed to variation in material quantities. By analysing the diffraction patterns shown in figure 2*f*, we were able to estimate the crystalline : amorphous ratios for these different samples, whose values range from *ca* 10% crystallinity in the 2% chitosan films to *ca* 15% in the 12% chitosan films. These observations point out that this variability in crystallinity could be potentially leveraged during fabrication for the creation of macroscopic samples with programmed structural anisotropy. To test this hypothesis, a sample was excised from a large printed construct (figure 3*a*) and micro-diffraction patterns were acquired from a series of positions along two ribs running at 90° angles to one another (figure 3*e*, at regions labelled C and D). Diffraction patterns from the two ribs are shown in figure 3*b,f*, and in the main intense ring, greater diffraction intensity is observed along an axis indicated by the green dashed lines. The maxima are more easily seen in the corresponding ‘unrolled’ plots of figure 3*c,g*, and the integrated peak intensity shown as a function of azimuthal angle chi in figure 3*d,h*. These results demonstrate that the diffracting planes in the polymer crystallites exhibit preferred orientation parallel to the direction in which the rib was printed.

### 3.3. Finite-element simulations

To gain additional insight into the factors driving the often-non-intuitive deformations in our printed constructs, we developed a finite element model to investigate the effects of these processes across a wide range of different printing parameters. The inputs for this model were derived from the real-time drying behaviour of printed constructs, as follows. After printing of the acetic acid-solubilized chitosan, the material begins to dry while still anchored to the textured aluminium substrate. Since the top surface of the printed chitosan is exposed to the surrounding atmosphere, it dries at a much faster rate than the underlying material, which is in contact with the aluminium. This resulting partially constrained drying mode results in a bilayer-like design [67,68], consisting of a fully cured and rigid top surface and a hydrated underlayer. Once this bilayer-like form is removed from the underlying substrate, the more hydrated underlayer begins to rapidly shrink, resulting in the induction of curling within the structure. It is important to note that despite the fact that the print direction could still be partially seen in the resulting additively manufactured forms owing to subtle variations in pigmentation (figure 4, bottom), the final surface morphology appears largely unstructured, suggesting that the folding dynamics could not simply be attributed to the presence of surface ridges formed during the printing process (which were not observed owing to immediate local material spreading, following extrusion).

In our simulations, the forms were similarly modelled as a bilayer architecture using the finite-element analysis software package, Abaqus (see §2 section for additional modelling details). Initial experiments of printed square samples of chitosan demonstrated a distinct dependency of the printing direction on the induced direction of folding, which we then modelled using orthotropic shrinking properties of the bottom layer. The auto-folding dynamics are consistent across both sheets (i.e. square samples in figure 4) and lattice geometries (such as those shown in figure 1).

Figure 4 shows the simulation results investigating the effects of structural anisotropy on the induction of predictable curling behaviour in a simple two-dimensional bilayer construct. In the absence of structural anisotropy, the square bilayer constructs exhibit an induced curling along either one of the short axes and never along the diagonal, with minute (and largely uncontrollable) differences driving this unpredictability in the folding direction. In contrast, we observed that for a high level of anisotropy in shrinkage (i.e. the material shrinks mostly in the direction parallel to the printing direction), there is a nearly linear relationship between the folding direction and printing direction.

More specifically, when the shrinkage ratio is *≥* $\frac{\surd 2}{2}$ , there is a sudden transition at *θ=* $\frac{\pi }{4}$ , such that as shrinkage becomes more uniform, only the two folded states along the short axes of the square are observed. These results demonstrate that for a square sample, a small imperfection can be amplified to fold the sheet towards one of these two possible edge-folding states, an observation that is in accordance with existing literature [69,70]. In contrast, and when comparing our simulation (figure 4, top) with our experimental results (figure 4, bottom), we are clearly capable of printing samples that fold along their diagonal, demonstrating that the difference in shrinkage in the directions parallel and orthogonal to the printing direction are $\frac{y-x}{L}$ *≤* $\frac{\surd 2}{2}$ . Since the direction of curling in our samples can be directly specified by the printing direction, these results suggest that it is indeed the manufacturing process that is responsible for induced anisotropic behaviour at the macroscale. While in the printed samples shown in figure 4, there are some subtle macroscopic features remaining (i.e. the low profile surface ridges, which were intentionally included here to better illustrate the print direction), similar folding behaviours were observed in printed samples, where these parallel surface features were significantly less prominent or virtually non-existent. Additional experiments using cast chitosan films of similar composition and geometries never exhibited diagonal folding, and furthermore, the direction of edge folding was random and not controllable, again suggesting a comparatively isotropic composition (compared to their anisotropic printed counterparts), an observation consistent with the relatively isotropic diffraction data shown in figure 1*a* (inset).

**Figure 5.**
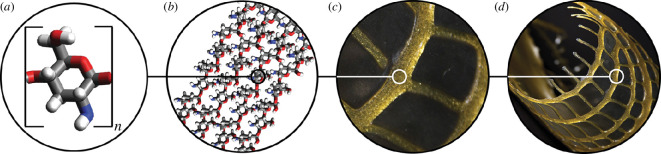
Schematic of the multi-scale behaviour model of our material system and fabrication strategy, where the induced orientation of nanoscale chitosan crystallites ultimately drives macro-scale folding of the final additively manufactured form. (*a*) Chitosan monomer, (*b*) acetic acid-solubilized chitosan crystallites depicted following a structural rib orientation printed with the WDFP, (*c*) 2.5-dimensional-printed structural rib and (*d*) chitosan construct folded along the print direction.

Consequently, nanoscale order induced by residual stresses during material extrusion in conjunction with the local and regional geometric design of tool-pathing logic could be leveraged to induce the observed large-scale folding patterns (figure 5), potentially decoupling global geometric design from induced material behaviour. As a result, for the same design, different material responses could be programmed such that global line paths could contain local and regional toolpaths within principal, secondary or other directions determining material alignment, which could be employed to direct specific folding patterns across different length scales.

## 4. Discussion

In the present study, we demonstrate the production of fully biodegradable [56] large-scale additively manufactured structures with anisotropic folding behaviour, using a single biopolymer in an aqueous form, at different concentrations and at ambient conditions. We additionally show that by altering the chitosan concentration, we can control the extent of material crystallinity within the printed material, with preferred crystallographic orientation dictated by the print direction. Through a combination of experimental and simulation-based approaches, we further demonstrate that this induced structural anisotropy can be leveraged to induce folding/bending of the resulting printed constructs in non-intuitive directions in otherwise geometrically planar structures. By combining this induced anisotropy of the chitosan crystallites and the introduction of residual stresses from the printing of a bi-layer-like construct [67,68], with small and large-scale venation patterns that exhibit three-dimensional structural relief [71–74], such as those shown in figure 1, this technology lays the groundwork for the design of hierarchical architectures that offer different length scale-specific and tunable folding responses. If we compare our work’s application space to other modern manufacturing processes inducing material or geometric anisotropy [3,48,57,75,76], our system could be useful when implemented at large scale, within difficult-to-monitor environments, and with limitations for use of simple or single materials. While other work on inducing orientation via additive manufacturing has focused on the use of magnets and magnetic additives within inks [34], on folding of shaped hydrogels within water baths [33], or in integration of fibrous fillers within blends [46] for the production of small-scale prototypes, several of these processes are difficult and potentially expensive to robustly scale up.

### 4.1. Future work

Derived from our work, the implementation of higher computational capacity predictive folding software could enable the rational design of much more complex geometries as extrusion-based structures with non-intuitive three-dimensional outcomes. The use of smaller inner diameter nozzles could improve resolution of the system with adequate reduction of material viscosity and/or increased extrusion pressure. Additional research combining low-concentration cast with high-concentration extruded material could also combine amorphous and ordered programmed behaviours for the emergence of functionally graded anisotropy. The introduction of reversible self-shaping of resulting structures could also be induced with the use of additives. Coating of structures with sustainable water-resistant compounds or the secondary cross-linking of the extruded biopolymers could also be employed in future iterations to make the resulting constructs more stable in their final folded forms and prevent further dehydration and decay. Finally, we envision that the contributions demonstrated by this work could ultimately be adapted for use in other polymer-based applications that require simple and robust solutions to produce large-scale complex forms. The potential for the more widespread implementation of the design principles described here is because our results are obtained from extrusion-based fabrication along with designed 2.5-dimensional toolpath directionality. The combination of these two simple methodologies induces nanoscale alignment and macro-scale folding without the need for multiple materials and additives, or of complex material curing devices during and after fabrication [14,17,18,22,77].

## Data Availability

Supporting information and additional experimental details are available from the authors on request.
